# Characteristic Genes and Immune Infiltration Analysis for Acute Rejection after Kidney Transplantation

**DOI:** 10.1155/2022/6575052

**Published:** 2022-11-02

**Authors:** Zechao Lu, Fucai Tang, Zhibiao Li, Zhixin Xie, Hanxiong Zheng, Jishen Zhang, Yanchun Gao, Zechu Lu, Yueqiao Cai, Yongchang Lai, Zhaohui He

**Affiliations:** ^1^Department of Urology, The Eighth Affiliated Hospital, Sun Yat-sen University, Shennan Zhong Road #3025, Futian District, Shenzhen, Guangdong 518033, China; ^2^The Second Clinical College of Guangzhou Medical University, Guangzhou, Guangdong, China; ^3^The First Clinical College of Guangzhou Medical University, Guangzhou, Guangdong, China

## Abstract

**Background:**

Renal transplantation can significantly improve the survival rate and quality of life of patients with end-stage renal disease, but the probability of acute rejection (AR) in adult renal transplant recipients is still approximately 12.2%. Machine learning (ML) is superior to traditional statistical methods in various clinical scenarios. However, the current AR model is constructed only through simple difference analysis or a single queue, which cannot guarantee the accuracy of prediction. Therefore, this study identified and validated new gene sets that contribute to the early prediction of AR and the prognosis prediction of patients after renal transplantation by constructing a more accurate AR gene signature through ML technology.

**Methods:**

Based on the Gene Expression Omnibus (GEO) database and multiple bioinformatic analyses, we identified differentially expressed genes (DEGs) and built a gene signature via LASSO regression and SVM analysis. Immune cell infiltration and immunocyte association analyses were also conducted. Furthermore, we investigated the relationship between AR genes and graft survival status.

**Results:**

Twenty-four DEGs were identified. A 5 gene signature (CPA6, EFNA1, HBM, THEM5, and ZNF683) were obtained by LASSO analysis and SVM analysis, which had a satisfied ability to differentiate AR and NAR in the training cohort, internal validation cohort and external validation cohort. Additionally, ZNF683 was associated with graft survival.

**Conclusion:**

A 5 gene signature, particularly ZNF683, provided insight into a precise therapeutic schedule and clinical applications for AR patients.

## 1. Introduction

Renal transplantation can significantly improve the survival rate and quality of life of patients with end-stage renal disease, and its effect is better than that of renal replacement methods such as chronic hemodialysis [1]. Although the graft survival rate has been significantly improved with the improvement of transplantation technology and the introduction of immunosuppressants [2], the probability of acute rejection (AR) in adult renal transplant recipients is still approximately 12.2%, which is not completely avoided [3]. Clayton et al. found that the early onset of AR would reduce the survival rate of grafts, resulting in the failure of transplantation and increasing the risk of death due to cancer and cardiovascular diseases [4]. At present, immunosuppressive regimens are often used in clinical treatment to prevent rejection after renal transplantation, which greatly improves the survival rate of renal transplant patients and grafts [5]. However, the long-term survival rate of transplanted kidneys has not improved [6]. AR is still an important factor leading to poor long-term prognosis in patients after renal transplantation [4]. In the early stage, AR is still reversible. Early prediction and early prevention of AR are conducive to preventing graft dysfunction and improving the prognosis of patients after transplantation [7]. Therefore, it is necessary to screen the characteristic genes of AR after renal transplantation.

Studies have shown that machine learning (ML) is superior to traditional statistical methods in various clinical scenarios [8]. Data-driven technology based on ML can use a large data repository to identify new risk gene signatures and more complex interactions between them to improve the performance of risk prediction [9]. Different studies have developed prognostic models for different types of cancers, such as glioblastoma [10] and hepatocellular carcinoma [11], through multiple ML techniques to predict the prognosis of patients and help clinicians screen potential responders for more targeted treatment [12]. The above results show that the disease signature genes based on a machine learning method to explore specific biomarkers have great potential. The support vector machine recursive feature elimination (SVM-RFE) algorithm is a method of recursively removing genes based on the weight of the support vector machine classifier (SVM) [13]. The SVM-RFE algorithm has been applied to genomics [14], which proves its strong performance. In multiple studies, ML has been used to investigate specific biomarkers associated with AR [7, 15, 16]. However, because the current AR study is only through simple difference analysis or a single queue, which cannot guarantee the accuracy of prediction, we intend to find a more accurate AR gene signature through ML technology and multiqueue verification.

In this study, we not only tried to find and validate some machine learning-based AR genes after renal transplantation, but also to explore their association with graft loss after renal transplantation. In summary, this study identified and validated new genes that contribute to the early prediction of AR and the graft survival after renal transplantation.

## 2. Materials and Methods

### 2.1. Data Collection

For this study, raw gene expression data were downloaded from the Gene Expression Omnibus (GEO) database (https://www.ncbi.nlm.nih.gov/) database. After screening, we finally adopted the three datasets GSE112927, GSE131179, and GSE21374 as research datasets. Among them, GSE112927 was used as the main research dataset. To improve the accuracy, 60% of the samples are used for training, and 40% of the samples are used for internal verification. We also selected GSE131179 as the external test dataset. GSE21374 is a dataset with clinical data used to further verify whether the selected genes have an effect on graft survival.

### 2.2. Differentially Expressed Genes Screening

The “limma” R package was utilized to the differentially expressed screen genes (DEGs) between AR and NAR in the expression data. Limma is an R package for analysing gene expression microarray data, specifically designed experiments using linear models and assessing differential expression. Taking [log fold change (FC)] > 1 and *P* < 0.05 as the filter value, the eligible DEGs were used for the next analysis. The DEGs we screened were visualized by heat map and volcano plot, which was generated by the “pheatmap” and “ggplot2” packages.

### 2.3. GO, KEGG, and GSEA Enrichment Analyses

To investigate the significantly enriched functions of DEGs between AR and NAR in GSE112927 and to better understand the important pathways involved in DEGs, the Gene Ontology terms (GO) [17], Kyoto Encyclopedia of Genes and Genomes (KEGG) [18], and Gene set enrichment analysis (GSEA) [19] were performed and analysed by using the “ggplot2”, “clusterProfiler”, “org.Hs.e.g.db”, “enrichplot”, and “GOplot” packages in the R package to further visualize the enrichment results. Among them, GO and KEGG analyses were performed using a cluster diagram, and GSEA marks the corresponding results of NAR and AR.

### 2.4. LASSO Analysis and SVM Analysis of the Obtained DEGs

The DEGs were screened by the LASSO and SVM [14] algorithms. To filter the characteristic genes, the “glmnet” package was used to perform regression analysis on the target genes. At the same time, support vector machine (SVM) was utilized to construct classification analysis by the“e1071”, “kernlab”, and “Caret”packages. Finally, the Venn package was used to determine the intersection of the characteristic AR genes in the two algorithms. Finally, five AR characteristic genes were obtained: ZNF683, EFNA1, HBM, THEM5, and CPA6. The “pROC” package was used to verify the disease-discriminating ability of characteristic AR genes in different cohorts. The role of these genes in graft survival was also investigated.

### 2.5. Immune Cell Infiltration and Immunocyte Association Analysis

CIBERSORT is a very commonly used method for calculating immune cell infiltration. It uses the principle of linear support vector regression to deconvolve the expression matrix of immune cell subtypes to estimate the abundance of immune cells. It is widely used to assess the types of immune cells in the microenvironment. It contains 547 biomarkers and 22 human immune cell phenotypes, covering plasma, B, T, and myeloid cell subsets. In this study, immune infiltration analysis was performed to determine which immune cells were mainly enriched in different graft survival groups.

### 2.6. Statistical Analysis

The “limma” R package was utilized for the DEGs. Heatmaps were generated based on the “pheatmap” package. By using the R package ggplot2 and other visualization data, the cluster graph was selected to generate the enrichment analysis graph of GO and KEGG, and GSEA enrichment analysis was also performed. AR characteristic genes were identified by LASSO analysis and SVM analysis. All statistical analyses were performed using R software. *P* < 0.05 was considered statistically significant.

## 3. Results

### 3.1. Screening DEGs in AR after Renal Transplantation

**Figure 1:**
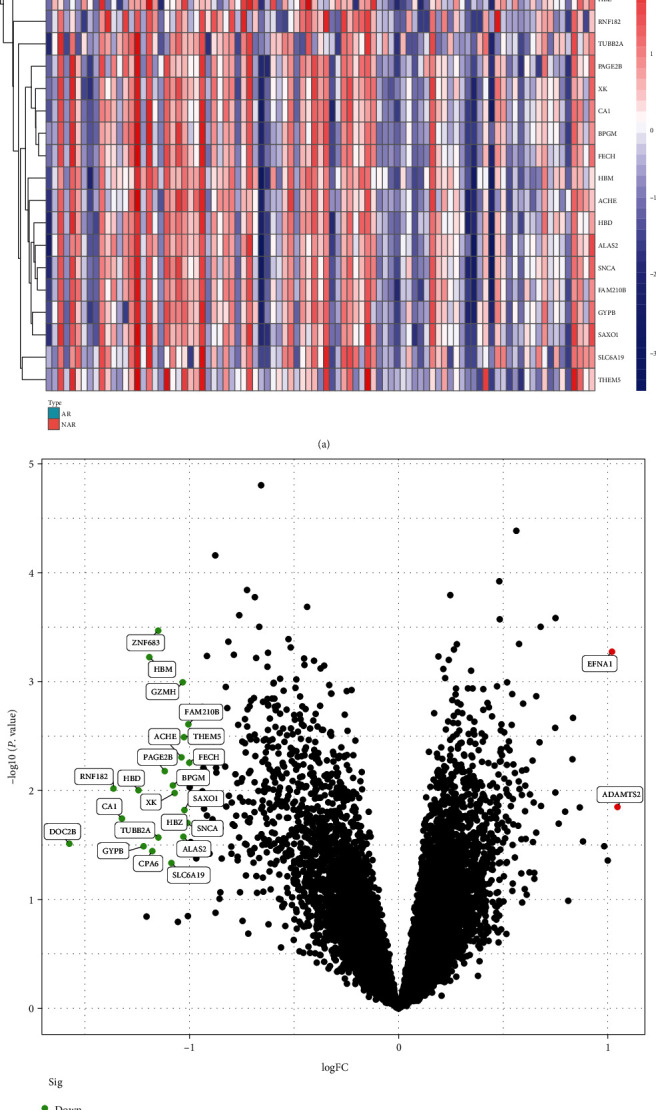
Screening for the DEGs (cut-off criteria of |log2FC| > 1 and *P* < 0.05). DEGs were visualized by heat map and volcano plots. (a) DEGs in the heat map. AR: acute rejection and NAR: nonacute rejection. (b) DEGs in volcano plots. Upregulated genes are indicated in red, and downregulated genes are indicated in green.

The cases of GSE112927 were divided into the training (*n* = 93) and the internal validation cohorts (*n* = 62). The training cohort in GSE112927 including 35 acute rejection (AR) cases and 58 nonacute rejection (NAR) cases was used to identify the DEGs. A total of 24 DEGs were identified: EFNA1 and ADAMTS2 were prominently upregulated, and 22 genes including ZNF683, HBM, GZMH, FAM210B, THEM5, ACHE, FECH, PAGE2B, BPGM, RNF182, HBD, XK, SAXO1, CA1, SNCA, HBZ, ALAS2, TUBB2A, DOC2B, GYPB, CPA6, and SLC6A19 were prominently downregulated (Figures 1(a) and 1(b)).

### 3.2. Analyses of Biological Processes and Pathways Enriched for DEGs

**Figure 2:**
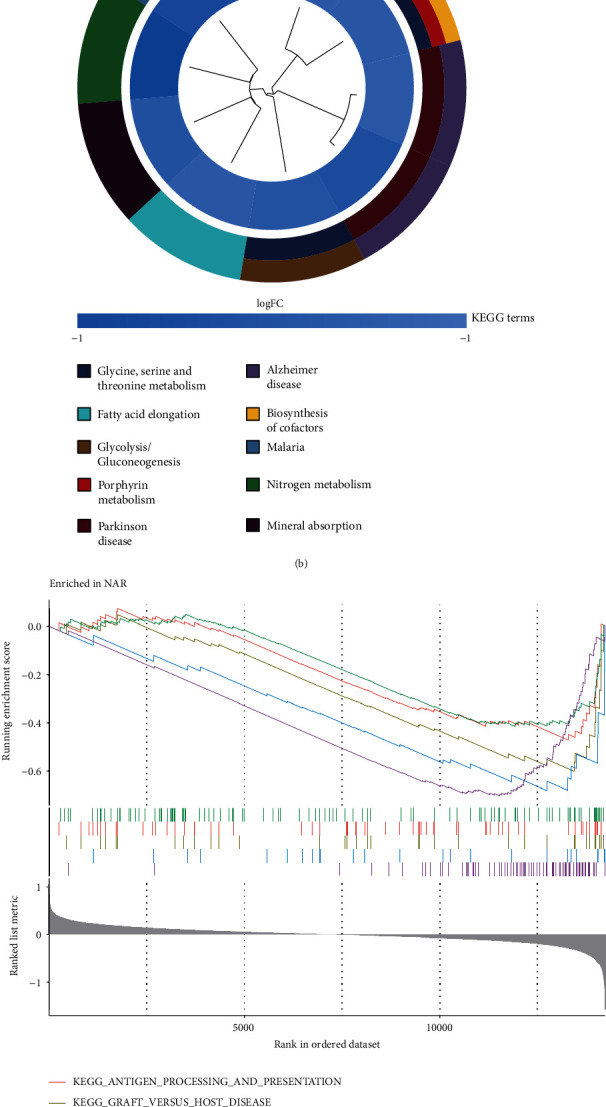
The DEGs were enriched using GO and KEGG functional enrichment analyses. (a) GO enrichment results. (b) KEGG enrichment results. (c) NAR enriched KEGG pathways using GSVA. (d) AR enriched KEGG pathways using GSVA.

Regarding the strength of our results, hydrogen peroxide catabolic process, mitochondrial matrix, haptoglobin binding, etc. were enriched GO items (Figure 2(a)). Glycine, serine, and threonine metabolism, porphyrin metabolism, biosynthesis of cofactors, etc. were enriched KEGG pathways (Figure 2(b)). Simultaneously, gene set enrichment analysis was applied to manifest the results corresponding to NAR and AR. According to the results, we found that five pathways, including antigen processing and presentation, graft versus host disease, natural killer cell-mediated cytotoxicity, porphyrin and chlorophyll metabolism, and ribosome, were substantially enriched in NAR (Figure 2(c)), while five other pathways, including calcium signaling pathway, complement and coagulation cascades, Ecm receptor interaction, neuroactive ligand receptor interaction, and starch and sucrose metabolism were enormously enriched in AR (Figure 2(d)). It is of profound significance for us to further understand acute rejection after renal transplantation.

### 3.3. Discerning the Characteristic AR Genes

**Figure 3:**
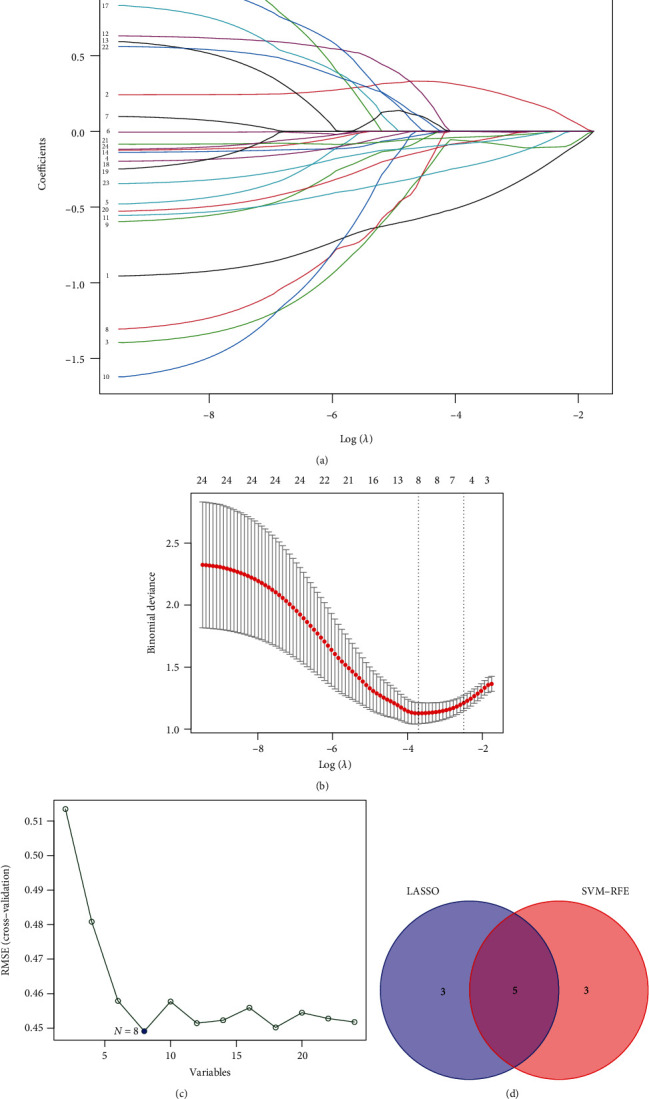
Selection of AR characteristic genes in kidney transplantation. (a, b) LASSO method. (c) SVM-RFE methods. (d) Venn diagram of common genes from the LASSO and SVM-RFE methods.

**Figure 4:**
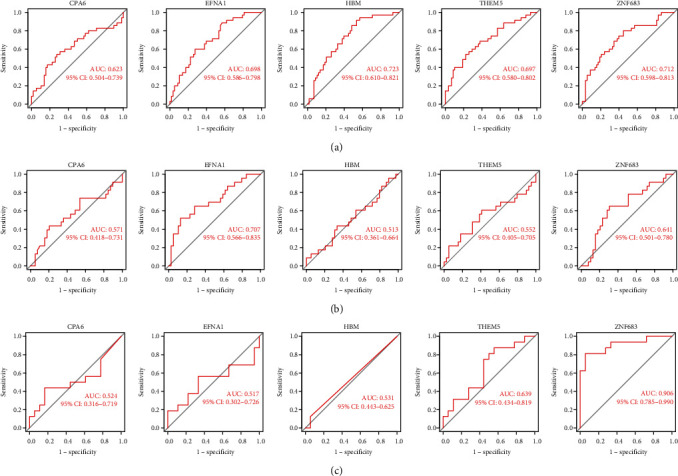
ROC of characteristic AR genes. (a) ROC plot of CPA6, EFNA1, HBM, THEM5, and ZNF683 in the training cohort. (b) ROC plot of CPA6, EFNA1, HBM, THEM5, and ZNF683 in the internal validation cohort. (c) The ROC plot of CPA6, EFNA1, HBM, THEM5, and ZNF683 in the external validation cohort.

To further select and validate the characteristic AR genes, we implemented LASSO regression analysis and SVM algorithm on the 24 DEGs mentioned above; CPA6, EFNA1, HBM, THEM5, and ZNF683 were screened by the two algorithms: LASSO (Figures 3(a) and 3 (b)) and SVM (Figure 3(c)). Furthermore, based on the training and internal validation cohorts in GSE112927 and the external test cohort in GSE131179, the five characteristic AR genes were assessed for their disease-distinguishing ability by plotting ROC curves. The results indicated that the AUCs of CPA6, EFNA1, HBM, THEM5, and ZNF683 in the training cohort were 0.623, 0.698, 0.723, 0.697, and 0.712 (Figure 4(a)), while the AUCs of these genes in the internal validation cohort were 0.571, 0.707, 0.513, 0.552, and 0.641 (Figure 4(b)). Finally, the AUCs of these genes in the external validation cohort were 0.524, 0.517, 0.531, 0.639, and 0.906 (Figure 4(c)). Only the AUC of ZNF683 was greater than 0.6 in the three cohorts. Therefore, it was the most critical gene in our following study.

### 3.4. Immunocyte Association Analysis

**Figure 5:**
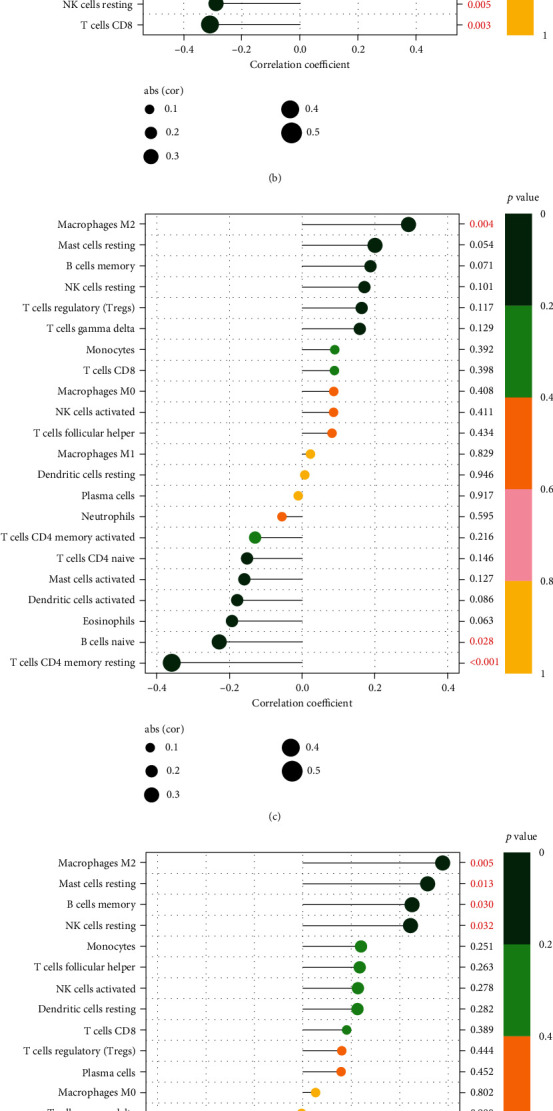
Association between characteristic AR genes and immune cell infiltration. (a–e) Correlations between AR characteristic genes and the immune cell infiltration level.

The Immunocyte association analysis results showed that T cells CD4 memory resting is a negative correlation with CPA6 (Figure 5(a)) while Macrophages M1 is the positive correlation. T cells CD8, NK cells resting, monocytes, and EFNA1 (Figure 5(b)) are negative correlated while B cells naïve, T cells CD4 memory activated, T cells gamma delta, T cells CD4 naïve, Macrophages M1, and EFNA1 are positively related. T cells CD4 memory resting and B cell naïve are correlated negatively with HBM (Figure 5(c)) and macrophages M2 is correlated positively with it. B cells naïve is negatively associated with THEM5 (Figure 5(d)) while macrophages M2, mast cells resting, B cells memory, and NK cells resting are positively associated with it. Finally, neutrophils, T cells CD4 memory activated etc. are negative correlation with ZNF683 (Figure 5(e)) while T cell CD8, NK cells resting etc. are a positive correlation. In the above mentioned results, we found that these five genes are associated with immune cells, and further research is needed to determine the specific mechanism.

### 3.5. Validation of the Effect of Characteristic AR Genes on Graft Survival

**Figure 6:**
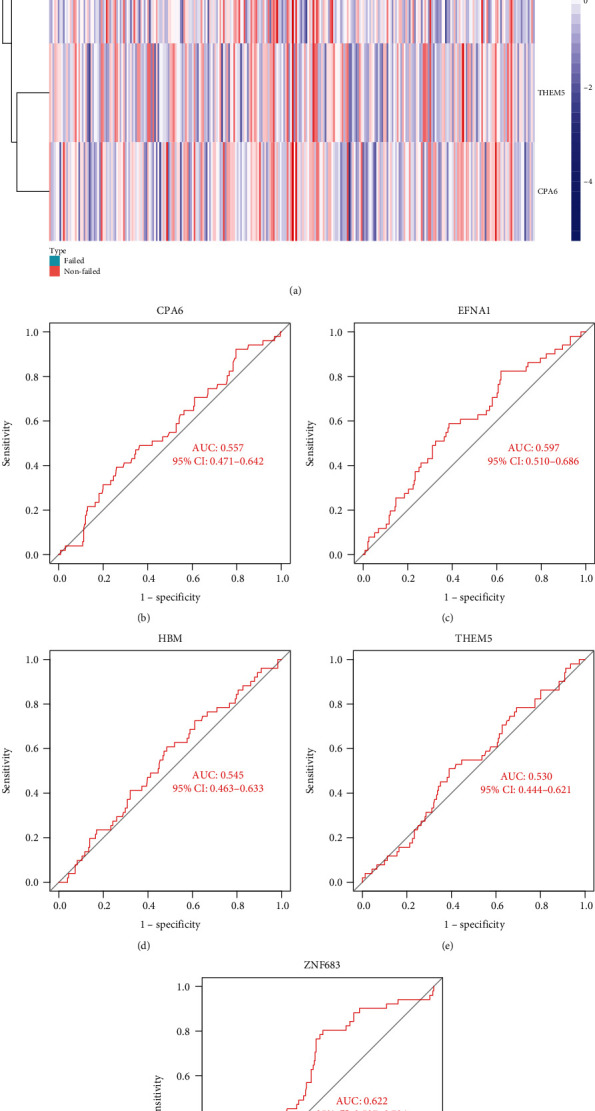
Validation of the effect of characteristic AR genes on graft survival. (a) Heatmap of characteristic AR genes. Nonfailed: Graft nonfailed. (b–f) ROC plot of CPA6, EFNA1, HBM, THEM5, and ZNF683 in GSE21374.

GSE21374 which contains the clinical data was used to further verify to whether the characteristic genes have any effect on graft survival. According to the results, we found that the graft survival analysis of ZNF683 (Figure 6(f)) was most meaningful in ROC curves, because its ability to distinguish failure of kidney transplantation was the best of the five genes. Therefore, we have a reason to identify it as the most critical key gene, and to study it further.

### 3.6. Graft Survival and Immunocyte Analysis of ZNF683

**Figure 7:**
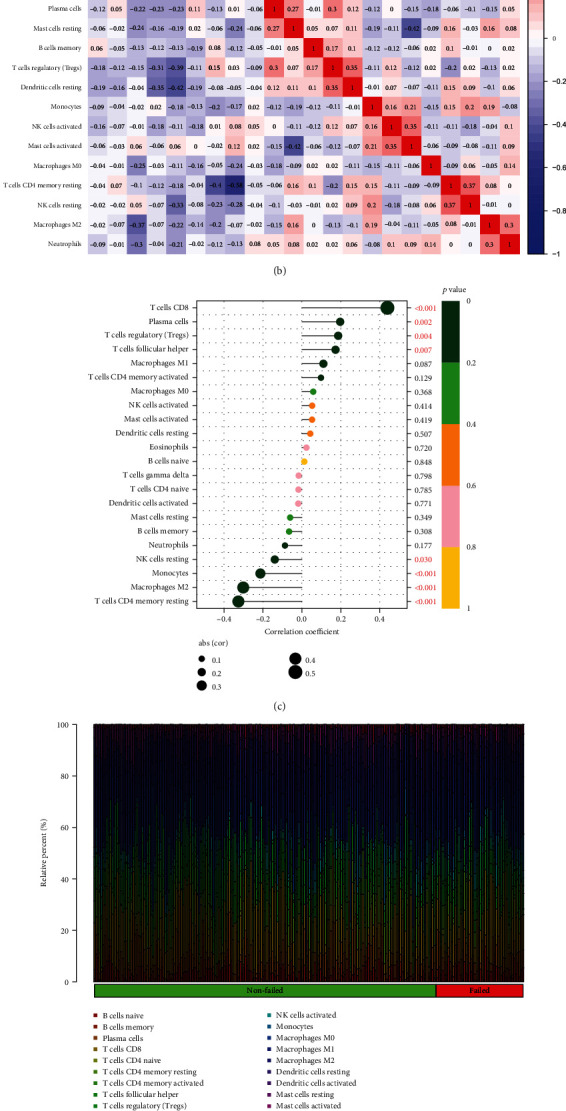
7 Graft survival analysis and immune infiltration analysis of ZNF683. (a) The ZNF683 gene graft survival curve. (b) Correlation analysis of immune cells. (c) The association between ZNF683 and immune cell infiltration in GSE21374. (d, e) The difference in immune infiltration between the graft failed and graft nonfailed controls. (*P* values < 0.05 indicated statistical significance).

The graft survival analysis indicated that the survival status in the ZNF683 high expression group was superior to that in the low expression (Figure 7(a)). Next, we utilized the corrplot package to carry out the correlation analysis of immune cells. For most immune cells, there is a negative regulatory relationship with each other (Figure 7(b)). We analyzed the association between ZNF683 and immune cell infiltration in GSE21374 (Figure 7(c)). Finally, we analyzed the difference in immune cells between Graft nonfailed and Graft failed groups. According to the result, we found that significant differences in the analysis of immune cells in nonfailed and failed groups are T cells CD4 memory activated, monocytes, and mast cells activated (Figures 7(d) and 7(e)).

## 4. Discussion

Despite important advancements in treatment regimens for kidney transplant patients, allograft rejection, especially acute rejection, remains a substantial threat and a leading predictor of allograft survival [20]. Therefore, the timely detection of acute rejection in advance of applying immunosuppressants can improve the survival rate of grafts after kidney transplantation [21]. The purpose of this study was to investigate genes associated with AR after kidney transplantation through a comprehensive analysis that will assist future continued searches.

In multiple studies, machine learning SVM has been shown to be an effective method for predicting kidney transplant prognosis. Mertens et al. identified and validated a set of 10 urinary protein biomarkers that can be used to exclude antibody-mediated rejection [22]. It has been reported that 5 genes can be used for the diagnosis and prediction of acute kidney injury after kidney transplant by weighted gene coexpression network analysis (WGCNA) [23]. The above study predicts kidney transplant prognosis in different ways from different molecules. However, machine learning SVM for finding disease-characterizing genes to predict acute rejection after kidney transplantation has not yet been discovered. In this study, a total of 24 differentially expressed genes were analysed, and five genes correlated with AR after kidney transplantation, including CPA6, EFNA1, HBM, THEM5, and ZNF683 were identified based on SVM and LASSO. According to the results of the ROC curve analysis, ZNF683 was screened out, which has not been reported in articles related to kidney transplantation. In addition, the gene ZNF683 was further explored in immune analysis, which may suggest that the gene ZNF683 serves as a biomarker of renal acute rejection.

In this study, multiple GEO datasets were used to identify characteristic AR genes after kidney transplantation. A total of 24 DEGs were obtained from the training cohort, including 2 upregulated genes and 22 downregulated genes. Based on two machine learning algorithms, 5 characteristic AR genes were identified. In particular, the AUC of ZNF683 ranged from 0.641~0.906, showing a high predictive performance for AR samples. The association between characteristic AR genes and immune cells was analysed. Based on the results, we found that Neutrophils, T cells CD4 memory activated, T cells gamma delta, T cells CD4 naive, and Macrophages M1 were negative correlation with ZNF683 while T cells regulatory (Tregs), NK cells resting, and T cells CD8 were the positive correlation. The zinc finger protein ZNF683 was originally termed the “homolog of Blimp1 in T cells” (Hobit). The gene was shown to regulate the development and maintenance of resident lymphocytes in innate tissue along with Blimp1, including tissue resident NK cells, tissue resident memory T cells, nature killer cells, and type 1 innate lymphoid cells [24–26]. Increasing evidence has showed that ZNF683/HOBIT is highly expressed in human effector-type CD8^+^ T cells, NK cells, and cytotoxic CD4^+^ T cells [27–29]. The gene was observed in cancer and organs affected by inflammation and viral infections [30–33]. A recent report showed that natural killer cells, natural killer T cells, CD4^+^ T cells, and CD8^+^ T cells were enriched in AR after kidney transplantation [34, 35]. In fact, CD8^+^ T cells are an important part of the cellular response in allogeneic rejection, and these cells recognize and bind MHC class I antigens, leading to allogeneic cell lysis and transplant rejection [36]. The infiltration of NK cells was associated with antibody-mediated rejection after kidney transplantation [35]. This is similar to our findings. In addition, a previously published report suggested that ZNF683/HOBIT also plays a key role in establishing tissue residency. The gene could maintain the numbers of T cells, inhibit the development of circulating memory cells and promote the formation and retention of tissue resident memory (TRM) cells [24]. The original donor TRM cells within the graft may have a positive effect on the preservation of organ transplantation. TRM cells can mount an immune response that counteracts rejection [37]. The persistence of donors TRM cells was shown to significantly improve patient graft survival in both bowel and lung transplants [38, 39]. Therefore, these findings suggest that ZNF683/HOBIT may maintain the number of the T cells and promote the number of the original donor TRM cells in kidney transplantation to protect the kidney from AR.

To further verify the role of the above AR characteristic genes in the kidney transplantation, survival analysis was performed using clinical data from GSE21374. Subsequently, the gene ZNF683 was further analysed. The graft survival of high ZNF683 expression was explicitly superior to that of low ZNF683 expression by the survival curve. The ZNF683 gene was significant and had a good effect on several ROC curves and was identified as a key gene.

However, the limitations of this study should be recognized. The information used in this research was downloaded from a public database. To further validate the ZNF683 gene in a prospective cohort, studies with larger sample sizes are required to validate our conclusions.

Taken together, the gene ZNF683 is an important gene to be discovered in kidney transplantation through machine learning, and its discovery may have a major impact on acute rejection after kidney transplant, which provides insight into a precise therapeutic schedule and clinical applications for AR patients.

## Data Availability

The gene expression and clinical data of AR patient samples were downloaded from the GEO (https://www.ncbi.nlm.nih.gov/geo).
